# Reading Words or Pictures: Eye Movement Patterns in Adults and Children Differ by Age Group and Receptive Language Ability

**DOI:** 10.3389/fpsyg.2017.00791

**Published:** 2017-05-22

**Authors:** Licong An, Yifang Wang, Yadong Sun

**Affiliations:** Beijing Key Laboratory of Learning and Cognition, College of Education, Capital Normal UniversityBeijing, China

**Keywords:** preschool children, receptive language ability, picture book reading, eye movement, adults

## Abstract

This study was conducted to explore the differences in the degree of attention given to Chinese print and pictures by children and adults when they read picture books with and without Chinese words. We used an eye tracker from SensoMotoric Instruments to record the visual fixations of the subjects. The results showed that the adults paid more attention to Chinese print and looked at the print sooner than the children did. The stronger the children’s receptive language abilities were, the less time it took them to view the pictures. All participants spent the same amount of time looking at the pictures whether Chinese words were present or absent.

## Introduction

Word awareness refers to an understanding of the forms and functions of words. Many scholars consider word awareness to be one of the most important predictors of the development of a child’s reading ability in early childhood (Ezell and Justice, 2000). Numerous studies have shown the close associations of word awareness with picture book reading and writing skills and the development of children’s reading abilities (Yaden et al., 1989; Bryant and Maclean, 1990). Children’s picture book reading, particularly, the fixation process, may reflect the development level of their word awareness, and an increasing number of researchers have started using eye movement technology to explore children’s picture book reading (Evans and Saint-Aubin, 2005; Justice et al., 2005). Eye movement analysis is currently one of the most effective methods for studying reading. Eye movement technology can record movements and collect a substantial amount of data throughout the reading process. Using data that we have captured using eye movement technology, we can conduct a sophisticated analysis of children’s reading and thereby objectively describe the features and rules of children’s picture book reading.

Picture books play an important role in the development of children’s word awareness. Picture books conform to the characteristics of the thoughts and interests of preschool children, and they have become popular reading materials. In addition, picture books are often used as research objects. For example, picture books have been used to improve children’s reading vocabulary (Evans and Saint-Aubin, 2013), to improve their early reading and writing skills (Schick, 2014), to cultivate their emotional literacy (Nikolajeva, 2013), to help them learn about gender roles (Anderson and Hamilton, 2005; Endendijk et al., 2014), to improve their understanding of false beliefs (Riggio and Cassidy, 2009) and to improve their environmental awareness (Hsiao and Shih, 2015). In contrast to books with both pictures and words, wordless picture books are unique in that they do not have a written narrative. Broadly defined, a wordless picture book has no text or has only one or two pages with text (Rothlein and Meinbach, 1996). Narrowly defined, a wordless picture book is a book with no text that tells a story that includes literary elements, such as themes, characters, a setting and a plot, which the author relates through a series of pictures (Russell, 1997). Picture books use mutually independent symbols of the iconic and the conventional to convey information (Nikolajeva and Scott, 2006). Nikolajeva and Scott (2006) use the term “circle hermeneutic” to describe the process by which children read picture books. In picture books, the text and pictures explain and supplement one another; if children process the pictures first, they form a new understanding of the text, and if they process the text first, they gain a more in-depth understanding of the pictures.

Numerous research studies have shown that the language abilities of monolingual children are good predictors of reading outcomes (Scarborough, 2001). Reading picture books is closely related to the receptive language abilities of children (Debaryshe, 1993; Fletcher and Reese, 2005). The primary characteristic of the eye movement patterns of children aged 4–6 years old is that as they age, their fixation counts on print increase; they also spend more time reading print and look at print sooner (Liu et al., 2011). Evans et al. (2009) observed a significant difference between the level of children’s fixations on a picture book and their receptive language abilities. The children’s receptive language abilities and letter knowledge explained 23–56% of the variance in the time that they spent viewing the print.

Most recently, researchers have found that preschool **c**hildren pay little attention to words (Justice and Lankford, 2002; Justice et al., 2005). Justice et al. (2005) studied the eye fixations of four American preschoolers aged 52–68 months as they read picture books. The authors observed that the average percentage of fixation counts on print was only 4% and that the average percentage of time spent looking at print was only 2.5%. Even when reading picture books with prominent text, the percentages of fixation counts and time spent on print were only 6 and 5.6%, respectively. Evans and Saint-Aubin (2005) studied five French-speaking preschoolers aged 48–61 months and focused on the fixation of the children’s eyes on the text when they read picture books. Their results indicated that, regardless of the type of picture books, the preschoolers fixed their gaze on the words only approximately 7% of the time. The researchers also used letter books with prominently highlighted text to study the visual fixation of preschoolers when reading. Their results indicated that the preschoolers’ visual fixation on illustrations clearly occurred earlier than their fixation on print and that the duration of their fixation on illustrations was clearly longer than that of their fixation on print.

Picture book reading is an important activity for preschool children. In studies that researchers have conducted in other countries, most of the test materials are in English, and the test results for the English environment may not predict the reading situation with regard to Chinese children. While several studies have documented skilled readers’ eye movement patterns during the reading process, few have examined group-level differences in fixations on print and pictures (e.g., children vs. adults) and the individual differences within a group. By examining the associations between children’s literacy skills and their fixations on Chinese print and pictures, such a study might help explain the mechanism behind age-related changes in fixations on print and illustrations. Based on existing research, we propose the following hypotheses: (a) Compared with adults, preschoolers pay little attention to the print when reading a picture book with Chinese words. (b) Preschoolers’ receptive language abilities are closely related to their eye movement patterns. (c) Adults and preschoolers spend the same amount of time looking at a picture irrespective of the presence or absence of Chinese words.

In the present study, we monitored the eye movement patterns of preschool children and adults to explore the differences in the degree of attention that two age groups (children vs. adults) paid to Chinese print and illustration as well as the individual differences within each different receptive language ability group when group members read picture books with and without Chinese words.

## Materials and Methods

### Participants

A total of 52 preschoolers, ranging in age from 54 to 78 months [mean (*M*) = 59.38; standard deviation (*SD*) = 21.74], from a kindergarten affiliated with Capital Normal University participated in this study, but we excluded seven children from the sample (six children had read the picture book before the experiment, and the eye movement data for one child were invalid). The final sample consisted of 45 children, and a total of 24 adults (7 males and 17 females), ranging in age from 22 to 26 years, from universities in Beijing. We used two picture books (one with words and one without) in this study. Participants were randomly assigned to read either a book with words (22 children, 12 adults) or a book without words (23 children, 12 adults). Basic information on the children is shown in **Table 1**.

**Table 1:** Characteristics of the participating children (*M*/(*SD*)).

Participant	With words	Wordless
*N*	23	22
Age (months)	61.30 (20.07)	57.36 (23.66)
Gender (boy/girl)	15/8	16/6
PPVT score	69.96 (20.17)	73.36 (17.49)
Literacy score	14.35 (9.21)	14.14 (10.47)

### Procedure

#### Literacy Sessions

First, the children were subjected to a literacy test. After repeated Chinese words were deleted, the Chinese words in the picture book were arranged randomly (see Appendix 2), and the experimenter asked the children to read the Chinese words one by one. The scores were recorded as 1 (correct) or 0 (incorrect). Only the children completed the literacy test (see **Table 1**).

#### PPVT Sessions

The next task was the Peabody Picture Vocabulary Test (PPVT), which was revised by Dunn (1965). This study used the version of the PPVT that had been translated and revised by the Shanghai Institute of Pediatrics. The PPVT comprises 120 sets of images and vocabulary, adding up to a total of 120 points. In this study, the test–retest reliability and the split–half reliability were both 0.945. Only the children completed the PPVT test (see **Table 1**).

Literacy is a sign of word awareness in children. Liu et al. (2011) found that the level of preschoolers’ literacy was significantly related to the level of their fixations on Chinese print. The receptive language abilities of preschoolers also affect the eye movement patterns of children when they read picture books. While we intended to use literacy scores as a predictor in the current study, we found that they were significantly correlated with PPVT scores (*r* = 0.492, *p* < 0.01), so we could not include both predictors in our study. Because other studies on reading use the PPVT, we opted to use this measure of general language ability rather than the literacy measure.

#### Eye Movements

**FIGURE 1:**
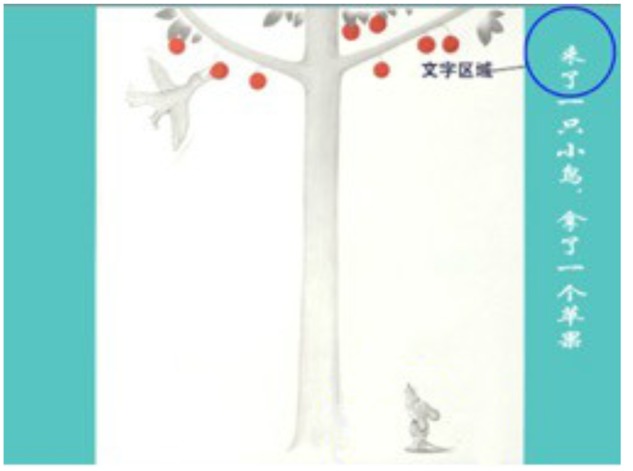
**Sample page from the picture book with words (the blue circle did not appear on the actual page)**.

**FIGURE 2:**
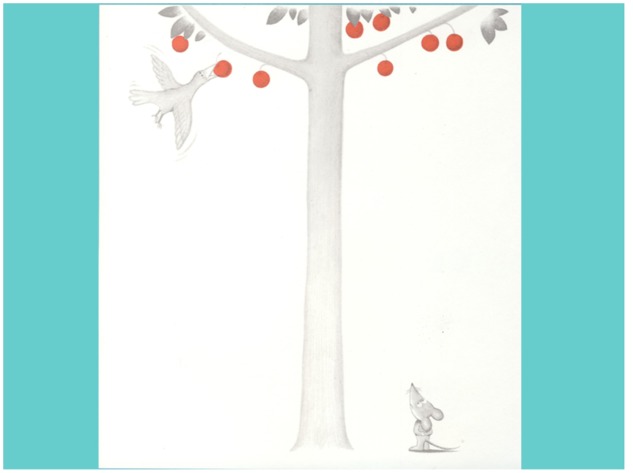
**Sample page from the wordless picture book**.

Eye movement experiments were conducted after the PPVT and literacy tests. The children were tested in a quiet room, and the subjects sat facing the experimenter. The participants first read a picture book on a computer screen; the book was irrelevant to the one that we used as the formal test book. The purpose of this task was to help the participants learn how to turn the pages by clicking the mouse (the adult subjects did not undergo this process); therefore, the children were allowed to proceed at their own pace. To create the experimental stimuli, we modified the storybook used for the formal experiment, Little Brother Mouse Wants an Apple (see Appendix 1). The clusters of text in the book ranged from 7 to 14 Chinese words in length. A total of 13 of the 16 pages were created as the experimental material. The 13 pages were scanned into pictures with an image size of 1024 × 768 pixels, which was equivalent to the resolution of the computer screen. Three of the 13 pages were not analyzed in the experiment. The home page was the cover, and the last page contained no Chinese words. The 12th page was structured very differently from the other pages. Therefore, those three pages were not included in the analysis of the eye movement data; the remaining 10 pages were included in this analysis. We used Photoshop to delete the words in the book to create a wordless picture book. However, we did not change the content of the book. Thus, we obtained two versions of the picture book, one with Chinese words (see **Figure 1**) and one without Chinese words (see **Figure 2**). Before the task, we asked the children to read the picture book without Chinese words, and we asked whether they could understand the book. This allowed us to determine whether the children could understand its content.

Each page of the picture book with words contained two areas of interest (AOIs) for fixation analysis: print and illustration. The print AOI consisted of several circles. An additional print AOI consisted of one circle (radius 75 pixels) around each Chinese print. The print AOI was fixed on the right-hand side of each page, but the blue circle around one word in **Figure 1** did not appear on the actual page. The entire picture on the page was an illustration AOI. The boundaries of the print AOI and the illustration AOI did not overlap.

The digital version of the book appeared on the monitor. The monitor’s resolution was 1024 × 768 pixels, and its refresh rate was 120 Hz. The subjects sat 60 cm away from the monitor. Our eye tracker for this experiment was model SMI-RED250, which SensoMotoric Instruments (SMI) produced as part of their high-speed series, and the sampling frequency was 250 Hz. Monocular data were recorded for the participants.

Next, we began the formal test. The monitor was positioned approximately 80 cm from the participants’ eyes. The participants could view the computer screen through a square window, which kept the children’s eyes focused on the stimuli. Subsequently, the experimenter conducted a 9-point calibration procedure.

### Dependent Measures

The analysis of dependent measures involved the following: the viewing time (the sum of all fixation durations in an AOI), the fixation counts (the number of fixation points in an AOI), the time to first fixation (the time period from when the participant began reading to when his or her gaze fell on an AOI for the first time), the proportion of fixation counts (the ratio of the number of fixation points on the target AOI to the number of fixation points on a page over a certain period of time), and the proportion of fixation length (the ratio of the fixation duration to the total viewing time).

The viewing time, the fixation counts, the proportion of fixation counts, and the proportion of fixation length related to the degree of attention given to the target (Justice and Lankford, 2002; Roy-Charland et al., 2007); when they increased, the degree of attention given to the target increased. The time to first fixation represented the participant’s early identification of the word; the shorter the time to first fixation was, the sooner the participant turned his or her attention to the target (Li et al., 2013).

**FIGURE 3:**
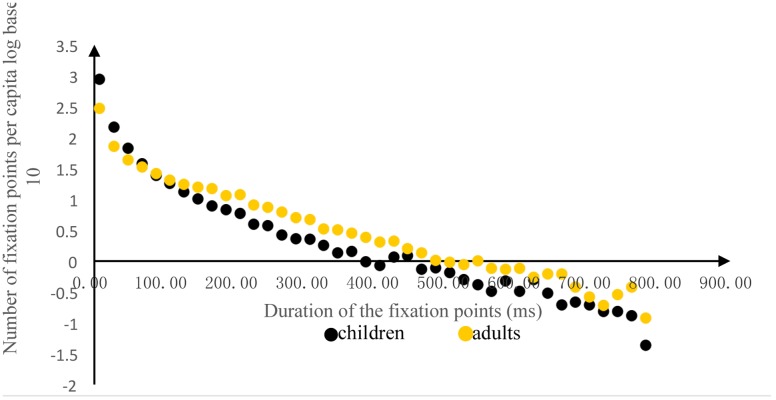
**Logarithmic distribution (log base 10) of the per-capita duration of different fixation points**.

We defined a fixation point as a fixation lasting longer than 100 ms, which is the standard that most studies of eye movement involving children’s picture books use (Salvucci and Goldberg, 2000; Nystrom and Holmqvist, 2010; Liu et al., 2011). **Figure 3** shows the logarithmic distribution (log base 10) of the per-capita duration of different fixation points. The duration of 100 ms was used as the point of demarcation. To the left of this point (i.e., fixation durations that were shorter than 100 ms), the children exhibited more fixation points than the adults, whereas to the right of this point, the opposite occurred. Using the duration of 100 ms as the standard, the fixation counts of the children were lower than those of the adults.

## Results

We calculated the correlation coefficients between the children’s reading skills (i.e., viewing time, fixation counts) and the PPVT scores for the entire book, the illustration AOIs and the print AOIs. For the entire book and the illustration AOIs, only the viewing time correlated negatively and significantly with the PPVT score (*r* = -0.37, *p* < 0.01; *r* = -0.45, *p* < 0.01). Other measures and PPVT scores were not correlated (i.e., fixation counts, time to first fixation) (all *ps* > 0.05). The results suggested that the higher the children’s PPVT scores were, the less time it took them to look at the book and to view the pictures. For the print AOIs, only the time to first fixation and the PPVT scores were negatively correlated (*r* = -0.49, *p* < 0.05); other reading skills were not correlated with the PPVT scores (i.e., viewing time, fixation counts) (all *ps* > 0.05), which illustrated that the children with higher PPVT scores looked at Chinese print sooner than those with lower PPVT scores.

**Table 2** presents the regression analysis, which was used to determine whether the children’s PPVT scores could explain the variance in their reading skills (i.e., viewing time, fixation counts) for the entire book, the illustration AOIs and the print AOIs. The results revealed that the PPVT scores accounted for 11% of the variance in the amount of time that children looked at the entire book, 18% of the variance in the amount of time that children looked at the pictures, and 20% of the variance in which children looked at the Chinese print first.

**Table 2:** Summary of regressions predicting reading skills.

AOIs	Dependent variable	Predictor	β	*t*	*R*^2^	*F*
Entire book	Viewing time	PPVT	-0.56	-2.58	0.11	6.63ˆ*
	Fixation counts		-0.24	-0.57	-0.02	-0.58
Illustration AOIs	Viewing time		-62.14	-3.29	0.18	0.84ˆ**
	Fixation counts		-0.05	-1.38	0.02	1.9
Print AOIs	Viewing time		14.04	1.03	0.00	1.07
	Fixation counts		0.04	1.08	0.01	1.13
	Time to first fixation		-19.01	-2.57	0.20	6.63ˆ*
	Proportion of fixation length		0.00	0.96	0.00	0.92
	Proportion of fixation counts		0.00	0.16	0.00	0.03

*PPVT, Peabody Picture Vocabulary Test*.

*^∗^*p* < 0.05; ^∗∗^*P* < 0.01*.

A 2 × 2 multivariate analysis of variance (MANOVA) using participant type (children vs. adults) and picture book type (with words vs. without words) as the independent variables was performed to compare the viewing time and the fixation counts with regard to the entire book and the illustration AOIs. Combined with the mean scores for dependent variables shown in **Table 3**, in terms of fixation counts for the entire book, the results showed that the main effects were significant [participant type, *F*(1,65) = 16.43, *p* < 0.01, η^2^ = 0.20; book type, *F*(1,65) = 15.95, *p* < 0.01, η^2^ = 0.20]. *Post hoc* comparisons [Tukey’s honest significant difference (HSD)] found that the adults had more valid fixation counts for the entire book than the children did. For fixation counts, the interaction between the picture book type and the participant type was significant. Simple effects revealed that adults’ valid fixation counts for the entire book were higher for picture books with words than for those without words [*F*(1,65) = 9.47, *p* < 0.01, η^2^ = 0.13].

**Table 3:** Mean scores (and standard deviations) of indices of each areas of interest (AOI) in picture books with and without words for each group.

AOIs	Indices	With words		Without words	
		Children	Adults	Children	Adults
Entire book	Viewing time	49513.48 (30389.91)	57251.67 (12947.70)	44774.09 (26483.28)	32376.67 (26367.46)
	Fixation counts	90.96 (65.34)	186.00 (41.72)	78.73 (34.46)	91.75 (62.32)
Illustration AOIs	Viewing time	3810.14 (2561.85)	3934.10 (1204.51)	4177.82 (2685.76)	2930.60 (2386.98)
	Fixation counts	7.19 (4.87)	12.72 (3.94)	7.29 (3.68)	8.22 (5.18)
Print AOIs	Viewing time	1141.20 (1288.86)	1791.16 (451.74)		
	Fixation counts	1.90 (3.15)	5.88 (1.48)		
	Time to first fixation	1114.31 (783.17)	936.87 (404.75)		
	Proportion of fixation length	0.21 (0.17)	0.32 (0.08)		
	Proportion of fixation counts	0.17 (0.16)	0.33 (0.09)		

For the illustration AOIs, we found that only participant type had a significant effect on the fixation counts [*F* (1,65) = 8.32, *p* < 0.05, η^2^ = 0.11]. However, other interactions and the main effects for the book type were not significant (*Ps* > 0.05). *Post hoc* comparisons (Tukey’s HSD) revealed that, compared with children, adults had significantly more valid fixation counts for the illustration AOI.

To compare the differences between the children and adults reading picture books, a single-factor analysis of variance (one-way ANOVA) using the participant type (children vs. adults) as the independent variable was performed to compare viewing time, fixation counts, the time to first fixation, the proportion of fixation counts and the proportion of fixation length with regard to print AOIs. Combined with the mean scores for dependent variables shown in **Table 3**, the results showed that the participant type effects were significant for fixation counts [*F*(1,33) = 16.99, *p <* 0.01, η^2^ = 0.34], the time to first fixation [*F*(1,33) = 9.82, *p* < 0.01, η^2^ = 0.24], the proportion of fixation counts [*F*(1,33) = 10.46, *p <* 0.01, η^2^ = 0.24], and the proportion of fixation length [*F*(1,33) = 4.86, *p* < 0.05, η^2^ = 0.13]. *Post hoc* comparisons (Tukey’s HSD) revealed that the adults looked at Chinese print sooner and that they had more valid fixation counts on the print AOI than the children did. In addition, the adults had higher proportions of fixation counts and fixation length on the print AOI than the children did (*p* < 0.05).

## Discussion

In this study, our main purpose was to examine the differences in the degree of attention that two age groups (children vs. adults) paid to Chinese print and illustrations as well as the individual differences within different receptive language ability groups when group members read picture books with and without Chinese words. The major findings of the present work are as follows. First, the adults paid more attention to Chinese print and looked at it sooner than the preschoolers did. Second, the stronger the receptive language ability of the children was, the less time it took them to view the pictures; in addition, the preschoolers with stronger receptive language abilities looked at Chinese print sooner than those with lower receptive language abilities. Third, the preschoolers and adults spent the same amount of time looking at a picture irrespective of the presence or absence of Chinese words.

### Attention to Words by Children and Adults

In this study, 33% of the adults’ fixation counts were on the Chinese print when reading picture books, and the corresponding figure for the preschoolers was 17%. The adults’ valid fixation counts for the print AOI were more than those of the children. These findings are consistent with prior research. Indeed, research has shown that children pay limited attention to words when reading (Justice and Lankford, 2002; Justice et al., 2005). The preschoolers’ average fixation counts for print account for only approximately 6% of the total fixation counts (Evans and Saint-Aubin, 2005; Justice et al., 2005; Evans et al., 2007, 2008). Compared with children, adults are mature readers and pay more attention to Chinese words—and do so sooner—when reading picture books with words, and they are more sensitive to Chinese print; by contrast, preschoolers have limited interest in Chinese words when reading independently.

According to the findings of this study, preschoolers with stronger receptive language abilities look at Chinese print sooner; this result suggests that preschoolers with stronger receptive language abilities intentionally look for Chinese words in books and that words are an important information source for these preschoolers as they attempt to grasp the meaning of a picture book. This finding is consistent with the results that previous researchers have reported (Roy-Charland et al., 2007). Roy-Charland et al. (2007) found that children paid more attention to the text as their grade level increased, and older preschoolers who were able to read all the storybooks spent more than half of their time fixating on the Chinese print. However, as preschoolers grow older, their receptive vocabulary also grows. All these phenomena can be expressed in terms of the development of word awareness. The stronger a child’s receptive language ability is, the higher his or her word awareness is and the more sensitive he or she is to words.

English words are composed of letters and syllables, while Chinese words are hieroglyphs that include a number of strokes packed into a square shape according to stroke assembly rules (Li et al., 2013); there is a close relationship between Chinese words and graphics. Compared with children from other countries, Chinese preschoolers appear to pay more attention to print. Han et al. (2011) showed that Chinese preschool children spent 19.9% of their total viewing time on the print AOI. The use of different picture books and the different divisions of print AOIs clearly reduce the comparability between research on Chinese subjects and that on subjects in other countries, which suggests the need for cross-cultural studies. For all forms of reading, researchers have found that the viewing time of Chinese children is longer than that of children from other countries.

### Attention to Pictures by Children and Adults

We also found that the stronger the children’s receptive language abilities were, the less time it took them to view the pictures. The preschool period is critical in the development of children’s image reading abilities, and children tend to primarily focus on illustration AOIs when they read (Justice and Lankford, 2002; Evans and Saint-Aubin, 2005; Justice et al., 2005; Evans et al., 2009). As preschoolers’ receptive language abilities continue to improve, the time that they spend processing illustrations gradually decreases (Sulzby, 1985; Roy-Charland et al., 2007; Gao, 2009). These previous findings coincide with those of the present study. These changes in children’s reading patterns may result from enhanced efficiency in image processing or from the conversion of images into available language (Li, 2011).

The type of picture book does not affect the level of fixation on pictures by preschoolers and adults. For pictures, the fixation duration and fixation counts of the preschoolers reading picture books with words are 3.8 s and 7.2 counts, respectively. For the preschoolers reading wordless picture books, the corresponding figures are 4.2 s and 7.3 counts, respectively. These results indicate that preschoolers’ interest in pictures does not decrease in the presence of Chinese words, and pictures still represent the main content in the reading of picture books. Preschoolers spend most of their time gazing at pictures. This conclusion also holds for adults. The relationship between text and images is a relationship of meaning, and text and images must be related through a similar or corresponding relationship, so that preschoolers integrate the picture and text after understanding each. Therefore, the addition of text does not reduce the extent to which preschoolers look at pictures.

### Limitations and Direction for Future Research

First, we use 100 ms as the minimum duration because most studies of eye movement involving children’s picture books, both in China and abroad, use 100 ms as the minimum duration (Salvucci and Goldberg, 2000; Nystrom and Holmqvist, 2010; Liu et al., 2011). However, the use of 100 ms as the minimum duration for illustration AOIs leads to higher fixation counts for adults than for children. Because the fixation duration for one point is generally shorter for children than for adults, children have shorter saccade amplitude (Rayner, 1978). Numerous studies use an 80 ms standard (Slattery et al., 2011). In studies of language processing, higher criteria may be beneficial because researchers have found that the vocabulary features of reading materials do not affect fixations of 140 ms or less (McConkie et al., 1992). In the future, we could investigate whether there are differences in children’s eye movement patterns for different fixation points.

Second, we also found that Chinese preschoolers appeared to pay more attention to Chinese print than do children who speak other languages to print in their native languages. Therefore, based on this study, we can conduct cross-cultural research to explore the influence of culture on children’s reading.

## Conclusion

The results of the present study reveal different patterns of eye movement in children and adults. As in previous studies (Evans et al., 2007), the adults looked at Chinese print sooner and paid more attention to it than the preschoolers did. All participants spent the same amount of time looking at the pictures irrespective of the presence or absence of Chinese print. The time to first fixation for the print AOIs and PPVT scores were significantly negatively correlated, and the viewing time of the illustration AOIs and PPVT scores were also significantly negatively correlated. These findings thus illustrated that the stronger the children’s receptive language abilities were, the more sensitive they were to the Chinese print and the less time it took them to view pictures.

## Ethics Statement

We conducted this study in accordance with the ethical standards of the Ethics Committee of the College of Education, Capital Normal University, and we obtained written informed consent from all subjects. We also obtained institutional review board approval for this study. Given that the subjects in this study were illiterate children, we received all the written informed consent forms from their parents. In the study, we performed all the procedures involving human participants in accordance with the ethical standards of the institutional and national research committees and with the 1964 Helsinki Declaration and its later amendments or comparable ethical standards.

## Author Contributions

LA, YW, and YS are all work and study in Beijing Key Laboratory of Learning and Cognition, College of Education, Capital Normal University.

## Conflict of Interest Statement

The authors declare that the research was conducted in the absence of any commercial or financial relationships that could be construed as a potential conflict of interest.
