# Supplementary material for: Reading Words or Pictures: Eye Movement Patterns in Adults and Children Differ by Age Group and Receptive Language Ability
Source: Front Psychol. 2017 May 22;8:791. doi: 10.3389/fpsyg.2017.00791 (PMC5439010; doi:10.3389/fpsyg.2017.00791)
Supplement: Supplementary file 1 [file Data_Sheet_1.docx]

**Appendix 1: Picture book**


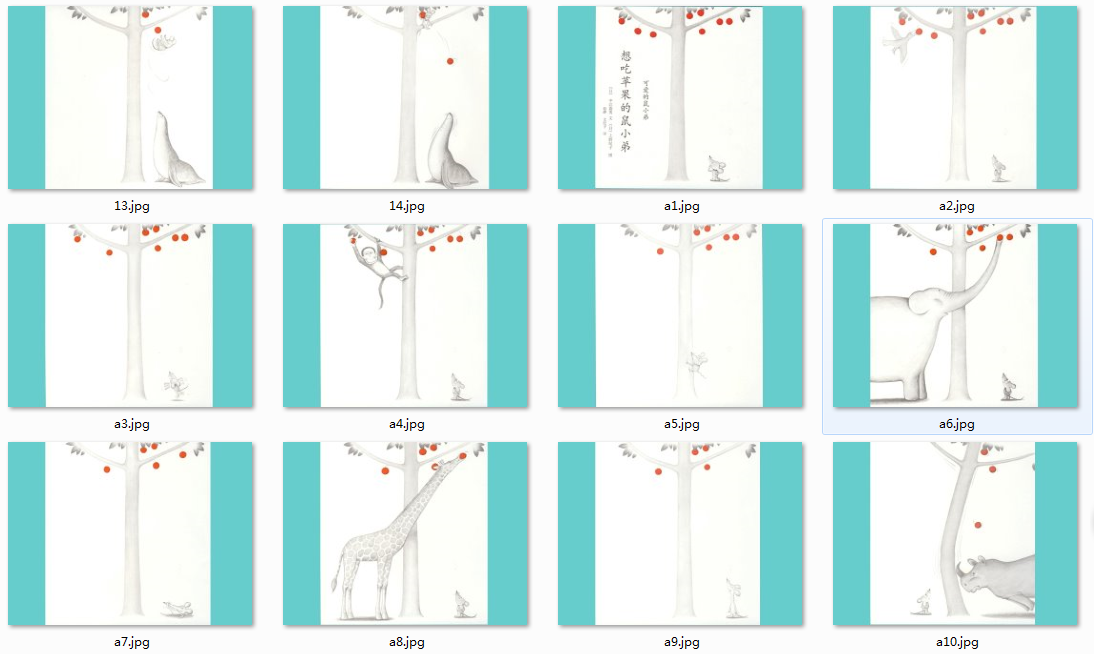


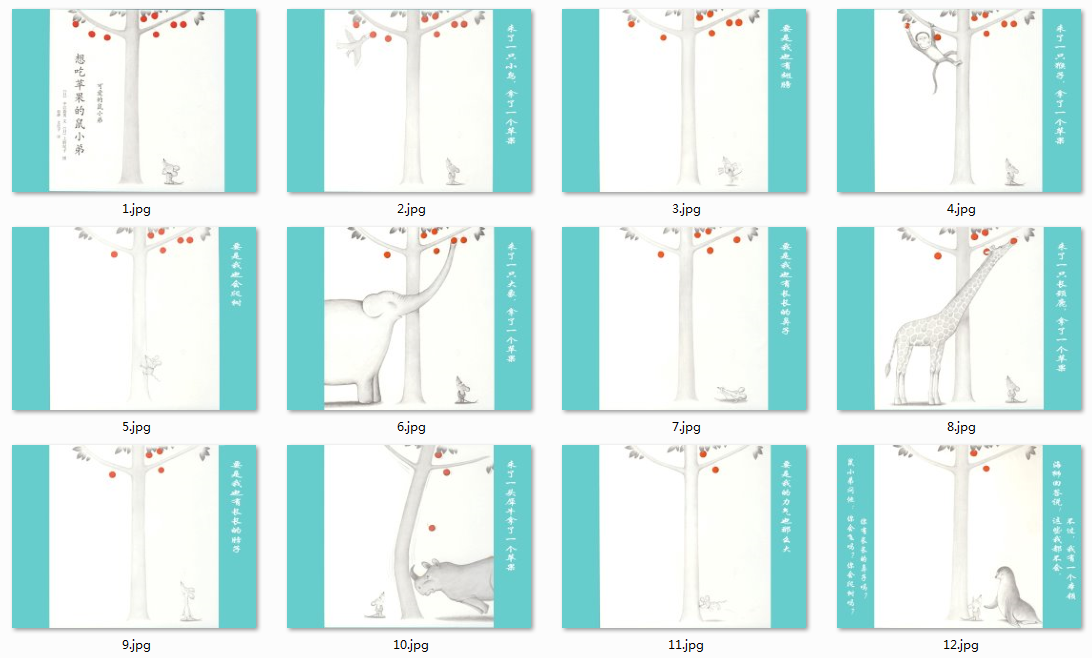


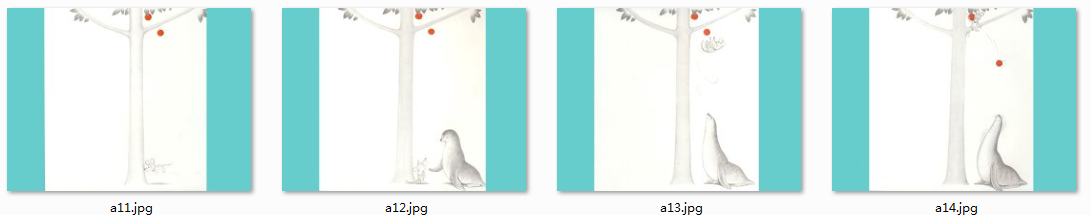


**Appendix 2: Printed text in the picture book**

| **爱** | **膀** | **鼻** | **脖** | **长** | **吃** | **翅** | **大** | **的** | **弟** |
| --- | --- | --- | --- | --- | --- | --- | --- | --- | --- |
| **猴** | **颈** | **可** | **来** | **了** | **鹿** | **拿** | **爬** | **苹** | **鼠** |
| **想** | **象** | **小** | **也** | **有** | **树** | **我** | **个** | **果** |  |
